# Supplementary material for: Complex spatiotemporal oscillations emerge from transverse instabilities in large-scale brain networks
Source: PLoS Comput Biol. 2023 Apr 12;19(4):e1010781. doi: 10.1371/journal.pcbi.1010781 (PMC10124884; doi:10.1371/journal.pcbi.1010781)
Supplement: Attachment — Submitted filename: response.pdf [file pcbi.1010781.s006.pdf]

Dear Editors,

We hereby resubmit our manuscript "Complex spatiotemporal oscillations emerge from transverse instabilities in large-scale brain networks" (PCOMPBIOL-D-22-01758). We truly appreciate your feedback on our manuscript, and thank the reviewers for their work. We appreciate the fact that both reviewers consider that our work presents interesting results and it is well written.

We have revised the manuscript in response to all comments and suggestions of the two reviewers. Below we present a detailed response to their remarks. Their original comments are highlighted in blue. All the changes in the manuscript are highlighted in red in a supplementary file.

Sincerely,

Pau Clusella, Gustavo Deco, Morten L. Kringelbach, Giulio Ruffini, and Jordi Garcia-Ojalvo

Response to reviewer 1

We thank the reviewer very much for their careful work and constructive comments on our manuscript, which we have addressed as follows:

1) Page 6, 15, banish -> vanish.

We have corrected the error.

2) Lyapunov exponents have dimension of inverse of time. Units should appear in Fig. 5(a), and in page 11 in the value of 1/lambda_1.

We agree with the reviewer that units would clarify the meaning of the exponents. We have now included them in the indicated locations.

3) Page 11, lines 34, I'd write m/s instead of ms^{-1}, to avoid confusion with the inverse of a millisecond.

We have implemented the suggestion.

4) Page 11. The formula for the mean resultant length appears to be incomplete.

We thank the reviewer for spotting this error, we have corrected the formula.

5) Page 25, I couldn't decipher equation (29).

Indeed, the formulation of the equation was wrong, since the summation was redundant. We have fixed the equation.

6) Page 26, I'd write down the formula of the Hilbert transform.

We now explicitly stated how the phase variables have been obtained, including the explicit formula for the Hilbert transform.

7) Equation (24) is inconsistent, as it equates a scalar to a vector.

We believe the reviewer is referring to Eq. (34), in which the modulus was missing. We have included it.

8) Same criticism for Eq. (35).

The modulus was also missing in this case. We thank again the reviewer for noticing these typos.

9) Eq (37), epsilon is already included in Eq. (36).

We have removed ϵ from Eq. (36) and left it in Eq. (37).

9) Equation (38). I_j stands for r_e.

The reviewer is right, we have corrected the equation.

Response to reviewer 2

We thank the reviewer very much for their careful work and constructive comments on our manuscript, which we have addressed as follows:

1) The synchronization of oscillations in NMMs has been investigated on simpler networks in previous works that I believe are quite relevant for the finding of the manuscript and should be cited. In early work, Ermentrout, G. B., and N. Kopell. "Multiple pulse interactions and averaging in systems of coupled neural oscillators." Journal of Mathematical Biology 29.3 (1991): 195-217 showed that excitatory coupling can lead to instability of in-phase synchronization. The variety of dynamical behaviors that ensue in a two node excitory-inhibitory networks was investigated in Borisyuk, G. N., et al. "Dynamics and bifurcations of two coupled neural oscillators with different connection types." Bulletin of mathematical biology 57 (1995): 809-840 including many periodic regime and a scenario for chaos. More recently, the stability of synchronized oscillations in a chain of oscillatory excitatory-inhibitory networks was investigated by Floquet techniques (the MSF of the present manuscript) in Kulkarni, A. et al "Synchronization, stochasticity, and phase waves in neuronal networks with spatially-structured connectivity." Frontiers in computational neuroscience 14 (2020): 569644. The instability of the two-node network at small coupling was reported to translate into a long-wave instability similarly to the “transverse instability” reported in Fig.2b of the present manuscript.

We thank the reviewer for pointing out to these relevant articles. We now cite them in the introduction and the discussion sections, and put them in the context of our work.

2) Importantly, in these previous works, it was found that the tranverse instability was quite sensitive to the type of synaptic connections between different network nodes. Even if one supposes, that different nodes connect by excitatory connections, it is a very strong assumption that the incoming excitatory connections only target the excitatory populations. In Kulkarni et al, it is in fact shown that the transverse instability disappears when the excitatory and inhibitory populations on a node are targeted in a similar fashion by the local and long-range excitatory connections. The authors should either convincingly argue why it is legitimate to neglect the long-range excitatory connections on interneurons or, better, investigate how their results are modified of they do not make this assumption.

Our modelling setup was inspired by the works from Forrester et al. (2020) and Kunze et al. (2015), which also consider long-range excitation targeting only pyramidal neurons. However, the reviewer is right that long-range excitation can also affect inhibitory neurons. We have performed a numerical exploration of this situation using the Jansen model (see Fig. 1 below), and did not find transverse instabilities. These new results are now mentioned in a dedicated paragraph of the discussion.

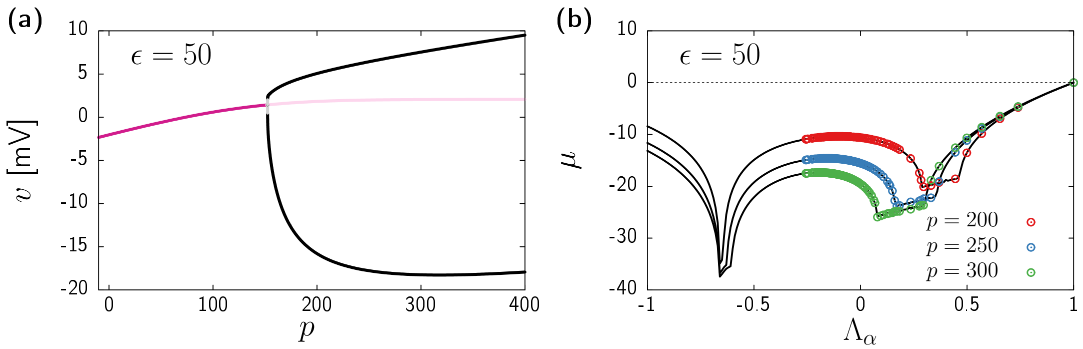

FIG. 1. (a) One-parameter bifurcation diagram of the homogeneous dynamics the Jansen model, with excitation targeting both pyramidal and interneuron populations (equations not shown). (b) Dispersion relation of the limit-cycle solution for different values of the external input p. No transverse instability originates for these parameter values.

3) Other issues that the authors should address, or at least discuss, is the biological realism of some of their other choices.

a) What is the impact of their neglect the local recurrent interactions between interneurons?

For the case of the Jansen model, we used the original parameter values of the system, and we did not find it necessary to change them without a specific reason. For the next-generation NMMs we took the parameters from Dumont et. al. (2019) and slightly changed them, which is why no recurrent inhibition is included. Nonetheless, to address the reviewer's valid comment we have explored whether setting $J_{\rm ii} = 10$ in the NG-NMM changes our findings. The results are presented in Fig. 2 below. As can be seen in that figure, the bifurcation structure of the homogeneous states in Fig. 2(a) is remarkably similar to that presented in the paper. We studied the presence of transverse instabilities for $\epsilon = 40$ and found positive Floquet exponents in this case (green shaded region in Fig. 2(b)). Similarly to what is presented in Fig. 7 of the paper, simulations in the transverse instability region lead to spatiotemporal dynamics (Fig. 2(c)). Therefore, we conclude that $J_{\rm ii}$ does not have a strong influence on the emergence of transverse instabilities. We now mention this in the revised manuscript when presenting the parameters in the "Next-Generation Neural Mass Model" subsection of the "Methods" section.

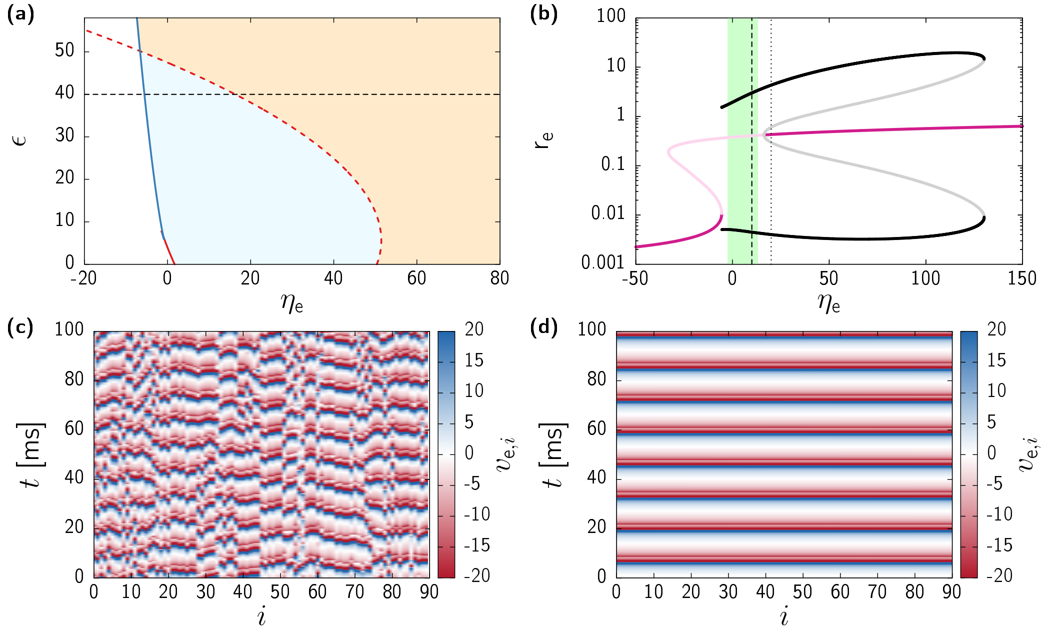

FIG. 2. Dynamics of the large-scale brain network given by NG-NMM. Same parameters as in the paper, except $J_{\rm ii} = 10$. (a) Two-parameter bifurcation diagram of the self-coupled NG-NMM system (Eqs. (40) of the paper). The continuous red line indicates a supercritical Hopf bifurcation, the dashed red line a subcritical Hopf bifurcation, and the continuous blue line a SNIC bifurcation. The horizontal black dashed line at $\epsilon = 40$ indicates the parameter range corresponding to panel (b). The light blue-shaded region indicates an area of stable homogeneous gamma activity. Finally, the orange-shaded region indicates bistability between homogeneous gamma activity and a fixed point. (b) One-parameter bifurcation diagram of the self-coupled NG-NMM system (Eqs. (40) of the paper) for fixed $\epsilon = 40$ (black dashed line in panel (a)). The green region indicates transverse instabilities of the limit-cycle solution. The vertical dashed and dotted lines at $p = 10$ and $p = 20$, respectively, indicate the values of the external input used in the simulations of panels (c) and (d). (c,d) Time evolution of each node as obtained from a numerical simulation of Eq. (38) of the paper for $\eta_e = 10$ (c) and $\eta_e = 20$ (d). In both cases $\epsilon = 40$.

b) An estimate of the coupling strength epsilon that one expects for the brain would also be welcome.

To the best of our knowledge there are no reliable estimations for global coupling strength in large-scale brain models. Typically these values are obtained by fitting the model to dynamical data, thus they depend on the model of choice. We explain this now at the end of the Discussion. However, we also note that the ϵ range where transverse instabilities emerge is within the range of the other coupling parameters of the Jansen model ($C_1, ..., C_4$), and therefore we expect that we are in reasonable parameter range. We now remark this within the subsection "Transverse stability" of the "Results" section.

c) If I understand well, the authors starts from a brain network with symmetric connections before row normalization. Moreover, they assume an identical NMM on each node. Other groups, and prominently X-J Wang's have argued otherwise. They found more appropriate to use Kennedy's data and moreover reported results consistent with a hierarchy of time scales in different areas as recently reviewed in Wang, X.-J. "Theory of the multiregional neocortex: large-scale neural dynamics and distributed cognition." Annual review of neuroscience 45 (2022): 533-560. These issues should be at least discussed.

The reviewer is tackling two different points here. First, regarding the symmetry of the structural connectome (prior normal-

ization), this topology is indeed symmetric, as most networks obtained from diffusion tensor imaging. However this is not a constrain to apply the MSF, which only requires the row-normalization step. This was briefly mentioned in the Methods section, and we have included a new sentence clarifying this point at the beginning of the Results section.

Second, regarding possible heterogeneities across brain regions. This is indeed an issue that we did not cover in our work, since we wanted to study a minimal setup. However, we believe that the insight gained from the analysis of a homogeneous network can help to understand the dynamics of heterogeneous models. We refer to this issue now in the Discussion.

d) Finally, it would be most welcome that the authors compare their results in a more detailed fashion to the available recordings. Are there features in their simulations that agree with what is found in the data (for instance, similar dephasing of oscillations in the model and the recordings). In this respect, alpha-waves in the brain usually do not appear as sustained oscillations but as transient oscillatory bursts. This is presumably not what is seen in the present model. This may suggest that the brain is actually functioning in a stable but fluctuating regime close to an oscillatory boundary. The authors should also discuss this issue.

This is certainly an excellent point. Comparing our results with real data would be indeed informative to validate some of the modelling assumptions made so far, and we have now clarified this in the Discussion. However, we believe that this should be explored in future work, as it goes beyond the scope of this article, which was meant to be focused on the analysis of transverse instabilities.

Nonetheless, we agree that some links between our current findings and relevant experimental literature should be emphasised. A comparison between the velocity propagation of alpha waves observed in our model, and those obtained in experimental studies was already present in the section "Periodic travelling waves". We have now added a new paragraph in the Discussion explaining the relevance of chaotic slow modulations of alpha activity to describe the turbulent dynamics observed in BOLD signals.

On the other hand, we agree that alpha oscillations might appear sometimes as transient bursts, for instance, during sleep. However, alpha rhythms usually emerge robustly and persistently in awake resting-state conditions, thus we believe that modelling alpha activity as self-sustained oscillation is appropriate in this case. We would also like to emphasize that we unveiled alternating activity between alpha and theta rhythms (see Fig5(c-e)). Exploiting this in other models where fixed-point and oscillatory activity alternate could produce further burst-like behavior. We have commented on this in the Discussion.

Minor concerns: - The authors should define the abbreviations they use (e.g. EEG, fMRI, NMM).

In the new version all abbreviations are defined on first use.
